# *kdr* mutations and deltamethrin resistance in house flies in Abu Dhabi, UAE

**DOI:** 10.1186/s13071-024-06128-5

**Published:** 2024-02-01

**Authors:** Mohamad Hamdan, Tamilarasan Kamalanathan, Asim Iqbal, Antony Raj Gnanaprakasam, Sabu Shajahan, Mohammad Hamad Alsadeq, Amgd sayed Ali, Mohammad Ali Al-Deeb

**Affiliations:** 1 Biology Department, UAE University, P.O. Box 15551, Al Ain, UAE; 2 Abu Dhabi Waste Management Centre (Tadweer), Abu Dhabi, UAE

**Keywords:** *Musca domestica*, Pyrethroid, Deltamethrin, *kdr*, Resistance, PASA, CDC bottle bioassay

## Abstract

**Background:**

The house fly, *Musca domestica*, is a significant carrier of diseases that can impact public health. Repeated use of pyrethroid insecticides may act as a selection pressure for mutations and amino acid substitutions in the house fly voltage-sensitive sodium channel (VSSC), which ultimately confers resistance. The objectives of this study were to determine the presence of knockdown resistance (*kdr*) mutations using molecular tools and to set up a CDC bottle bioassay specific for house flies in the United Arab Emirates (UAE) to screen for deltamethrin resistance.

**Methods:**

Adult flies were collected from 19 locations in Abu Dhabi, UAE, and DNA was extracted, followed by PCR amplification of specific alleles (PASA) and conventional PCR using several primers to amplify regions of the VSSC gene. Sanger sequencing was performed on PCR products. We also designed primers that detect four *kdr* mutations using complementary DNA (cDNA) in reverse transcriptase (RT)-PCR followed by Sanger sequencing. Additionally, a CDC bottle bioassay was set up for detecting deltamethrin resistance in adult house flies.

**Results:**

In PASA, the primers successfully amplified the target bands (480, 280 and 200 bp). The *kdr* allele was found in flies collected from 18 of the 19 locations, at the highest and lowest prevalence of 46.9% and 9.4%, respectively. Resistant homozygous (RR) insects constituted 5.0% of the tested populations, and heterozygous (RS) insects accounted for 36.5%. The RR genotype was prevalent in house flies collected at 10 of 19 sampling locations. House fly populations were mostly in Hardy–Weinberg equilibrium, except in three locations. In addition to verifying the presence of the previously identified *kdr* mutation L1014F, in this study we detected two *kdr *mutations, L1014H and T929I, that have not previously been reported in the UAE. Also, for the first time in the UAE, a CDC bottle bioassay for deltamethrin resistance was used, which found that 60 min and 4.5 µg/ml were the diagnostic time and dose, respectively. Using this assay, we detected deltamethrin resistance in house flies from two of 16 locations, with a resistance level of 12.5%.

**Conclusions:**

Using DNA sequencing, we confirmed the presence of a known *kdr* mutation and uncovered two new *kdr* mutations in house flies from Abu Dhabi. Additionally, we detected deltamethrin resistance in these flies using a CDC bottle bioassay. Further research is recommended to comprehensively identify more *kdr* mutations in UAE house fly populations and assess their impacts on control strategies.

**Graphical Abstract:**

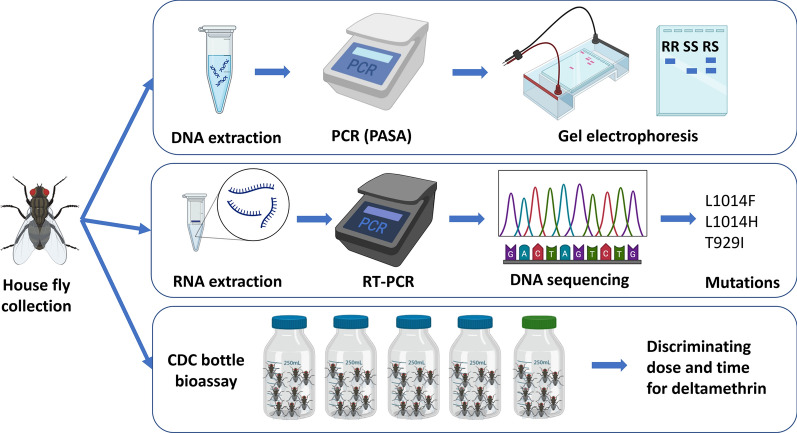

## Background

The common house fly, *Musca domestica* L. (Diptera: Muscidae), is a major carrier of diseases [1–4]. This versatile insect has co-evolved with human activity, making it well-suited to urban, suburban and rural environments [5]. House flies play an essential role in ecosystems by assisting in decomposing organic matter and aiding in nutrient cycling [6, 7]. Their rapid life-cycle, from egg to adult, enables them to establish populations and adapt to changing environments [8, 9]. House flies exhibit a remarkable skill for seeking nourishment in various food sources while also serving as carriers of microorganisms. Thus, they are potential health concerns for both humans and animals [10]. Due to their high mobility, they can transfer bacterial cells from heavily contaminated surfaces across various environments. The dynamics of host microbiota composition are shaped by influences such as environmental exposures, developmental stages, geographical locations and seasonal changes [11, 12]. Taking all of these factors together, house flies act as vectors for wide range microbes, and comprehending their role in influencing the distribution of bacteria and fungi within their surrounding environments, including human habitats, has significant potential for yielding valuable insights [13, 14].

 House flies can mechanically transmit pathogens by landing on surfaces and food items, depositing the microorganisms they carry [15]. Studies have shown that the combined effects of bacterial density have the potential to profoundly influence insect health, as modifications in microbial group composition frequently correlate with disease incidence [16]. House flies can transmit pathogens responsible for over 100 human and animal diseases [17]. The consistent exposure of flies to waste and animals establishes a prime opportunity for disseminating pathogens to human and animal populations. House flies have been identified as vectors for various pathogens, including *Campylobacter* spp. and *Shigella* spp. [18, 19], and carriers of bacteria, such as *Campylobacter jejuni* [20], *Salmonella* spp. [21], *Staphylococcus aureus*, *Pseudomonas aeruginosa*, *Enterococcus faecalis* [22] and *Escherichia coli* [23–26], underscoring their role in potential disease transmission [27]. Furthermore, they are carriers of serious pathogens responsible for a range of health issues, such as meningitis, foodborne illnesses, diarrhea, abscesses, bloodstream infections and hemorrhagic colitis, and thereby represent significant health concerns [28]. Their adaptability, mobility and affinity for human environments pose substantial health risks as carriers of various pathogens responsible for diseases in humans and animals. Thus, the management and control of house flies are crucial in terms of safeguarding public and animal health across the globe.

Several insecticides have been used in chemical control programs targeting house flies worldwide. In Abu Dhabi, United Arab Emirates (UAE), the management of house flies involves measures to control both larvae and adults. Larviciding includes the use of insect growth regulators (IGRs) and conventional larvicides. Toxic fly baits are one of such methods used for the chemical control of adult flies. Residual sprays are also employed against adult flies using long-lasting insecticides. Outdoor space treatment is only permitted in the case of high fly densities, disease outbreaks or potential disease outbreaks, and is standardly conducted using misting backpack machines, vehicle-mounted ultra-low volume machines or thermal fogging. In Abu Dhabi, deltamethrin, lambda-cyhalothrin, cypermethrin, cyfluthrin, tetramethrin, alphacypermethrin, cyromazine, thiamethoxam and imidacloprid are the most commonly active ingredients used for the chemical control of house flies [29]. However, the use of these chemicals has resulted in a rapidly growing house fly population that is producing offspring resistant to their effects, making house fly control through the use of these insecticides increasingly challenging [30].

 Physiologically, insect resistance manifests in several ways, including reduced insecticide uptake, enhanced detoxification mechanisms and alterations in the target sites of the insecticide [31]. In 1951, Busvine [32] documented the first non-metabolic resistance factor in house flies, denoted knockdown resistance (*kdr*), which reduces the effectiveness of pyrethroids and dichlorodiphenyltrichloroethane (DDT) insecticides. This resistance is due to mutations in the gene coding for the voltage-gated sodium channel (VGSC) protein, particularly the L1014F mutation [33]. Genetic factors, such as *CYP6D1* and *Vssc1* alleles, also contribute to pyrethroid resistance. A single amino acid substitution can change the response of the house fly's sodium channel [34, 35]. The L1014F *kdr* allele in house flies is confirmed to be a fully recessive genetic trait [36, 37]; this means that for an individual house fly to exhibit resistance to pyrethroid insecticides due to the L1014F *kdr* allele, it generally needs to inherit two copies of this allele (one from each parent). House flies with just one copy of the allele (heterozygous) are less likely to show the same level of resistance as those with two copies (homozygous). Using PCR amplification of specific alleles (PASA) analysis of house fly populations strongly suggests that the *kdr* mutation is a significant mechanism for pyrethroid resistance. In a study carried out in 2014 in the UAE which used PASA, some housefly populations were reported to have the L1014F mutation, highlighting the need for pyrethroid resistance management programs [38]. The L1014F mutation is commonly found when insect resistance is detected, but there are also other *kdr* mutations. Methionine-*kdr* house fly strains, which carry a mutation in which methionine is substituted by threonine at position 918, was found alongside the L1014F mutation in the sodium channel coding sequence [39]. Thus, the *kdr* mutation L1014F is not the only mutation reported in house flies. Several studies indicated that other mutations, such as *kdr*-his L1014H, *super*-*kdr* M918T and T929I, confer resistance to pyrethroid insecticides [40–42]. Overall, house fly resistance, notably *kdr* mutations, poses a significant challenge to achieving house fly population control through chemical control programs. Understanding the genetic and physiological factors and their geographical distribution is vital for developing effective management strategies.

To determine insecticide resistance in insects, scientists often conduct bioassays using either insecticide formulations or active ingredients. For example, in mosquitoes, resistance is commonly measured using the WHO protocol [43] or the US Centers for Disease Control and Prevention (CDC) bottle bioassay [44]. In house flies, the most commonly used method is the topical application bioassay, which has been employed in various countries [45, 46]. This method involves applying a known amount of an insecticide directly onto the surface of the fly, typically onto the thorax or abdomen. The CDC bottle bioassay, which can also be used to examine resistance in different insect species, not just mosquitoes, has been used in a number of studies in house flies [47, 48]. This method is known for being simple, cost-effective, field-compatible and capable of detecting even low levels of insecticide resistance.

The *kdr* mutation L1014F was discovered in house flies in the UAE in 2014 for the first time. However, there have been no studies in the UAE since 2014 aimed at assessing the status of this mutation in house fly populations. Collecting such data has significant importance due to the potential impact of *kdr* resistance on the efficacy of pyrethroid insecticides in controlling house fly populations. To address this knowledge gap, the aims of the study reported here, which focuses on the Emirate of Abu Dhabi, were to detect the presence of *kdr* mutations using molecular tools and to set up a CDC bottle bioassay specific for house flies in the UAE to screen for deltamethrin resistance. The outcomes of this research contribute to global knowledge on house fly resistance and have the potential to substantially enhance house fly control strategies in the UAE, thereby benefitting both human and animal health.

## Methods

### Insect collection

**Fig. 1:**
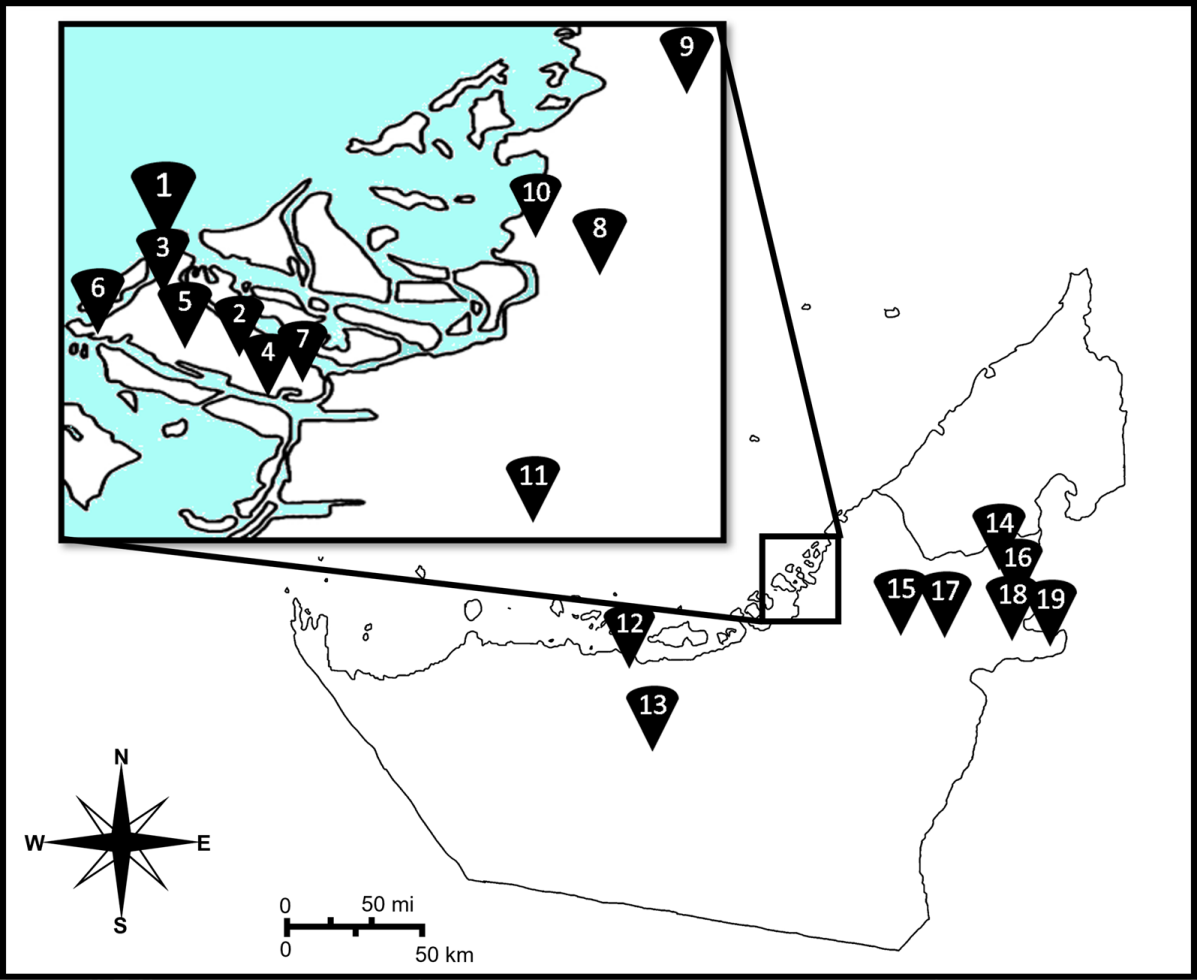
Map of United Arab Emirates showing the collection locations of the house fly *Musca domestica* in the Emirate of Abu Dhabi, 2023. 1, Mina Zayed; 2, Hadbat Al Zafranah; 3, Al Danah, 4; Al Muzoun; 5, Al Mushrif; 6, Al Bateen; 7, Al Rawdah; 8, Shahama City; 9, Al Samha; 10, Al Bahiya; 11, Baniyas; 12, Mirfa; 13, Madinat Zayed; 14, Al Hayer; 15, Al Khazna; 16, Hili; 17, Remah; 18, Central district; 19, Al Shuwaymah

House flies were manually collected using fly sweep nets in 2023. Once in the laboratory, they were counted, sexed and preserved in a - 20°C freezer. To ensure comprehensive coverage of the Abu Dhabi Emirate, the sampling was conducted in 19 locations, as listed and shown in Fig. 1.

### DNA and RNA extraction

A total of 279 female house flies were used for the PASA tests, and 18 randomly selected flies employed for the reverse transcriptase-PCR (RT-PCR) and Sanger sequencing. For genomic DNA (gDNA) extraction, the thorax of each individual house fly was excised and then homogenized using a bead homogenizer (Benchmark Scientific, Sayreville, NJ, USA). DNA was extracted from the homogenate using the DNeasy Blood & Tissue Kit (Qiagen, Hilden, Germany) following the manufacturer's recommended protocol. For the RT-PCR, total RNA was extracted from the thorax using the RNeasy Mini Kit (Qiagen) following the manufacturer’s protocol. The concentration of DNA and RNA in each sample was assessed using a NanoDrop 2000 UV spectrophotometer (Thermo Fisher Scientific, Waltham, MA, USA). The extracted DNA and RNA samples were preserved in a freezer at − 20 °C.

### PCR and Sanger sequencing

Utilizing the PASA protocol outlined by Huang et al. [34], we investigated the presence of the *kdr* mutation L1014F in the collected house flies. Two outer allele-specific primers, *kdr* 1 and *kdr* 4, together with two inner allele-specific primers, *kdr* 2 and *kdr* 3 (Table 1), were used to amplify the target regions. The PCR conditions mirrored those detailed in Al-Deeb [38]. Three separate PCR assays were run for each DNA sample (1 sample from each insect). Each these three PCR assays contained two primers: *kdr* 1, *kdr* 4; *kdr* 1, *kdr* 3;, and *kdr* 2, *kdr* 4. Following amplification, the DNA fragments were separated by electrophoresis in 1.5% agarose gels stained with ethidium bromide and photographed under UV light (omniDOC SAFE Gel Documentation System; Cleaver Scientific, Rugby, UK). Genotyping of each insect was achieved by interpreting the gels based on band size and quantity. Genotypes were classified as homozygous resistant (RR; *kdr*/*kdr*), heterozygous (RS; *kdr*/*sus*) and homozyous susceptible (SS; *sus*/*sus*). The *kdr* 1 and *kdr* 4 primers generated a control fragment (480 bp) on the gel while the *kdr* 1 and *kdr* 3 primers produced a 200-bp fragment, representing the susceptible allele. The *kdr* resistant allele was represented by a 280-bp fragment produced by the *kdr* 2 and *kdr* 4 primers. Consequently, the RR genotype displayed a single 280-bp band, the SS genotype exhibited a solitary 200-bp band and the RS genotype manifested both the 280- and 200-bp bands.

**Table 1:** Primers used for the detection of knockdown resistance mutations in house flies in Abu Dhabi, United Arab Emirates

Primer	Direction	Sequence 5′-3′	Primer size (bp)	Annealing temperature (°C)	Used for	References
*kdr* 1	Forward	AAGGATCGCTTCAAGG	16	54	PASA	[34]
*kdr* 2	Reverse	GTCGTGATCGGCAATT	16	54	PASA	[34]
*kdr* 3	Forward	CGTCAACTTACCACAAG	17	54	PASA	[34]
*kdr* 4	Reverse	TTCACCCAGTTCTTAAAACGAG	22	54	PASA	[34]
K1	Forward	TCGCTTCAAGGACCATGAAT	20	60	Sequencing	[47]
K2	Reverse	TTACGTTTCACCCAGTTCTTA	21	60	Sequencing	[47]
4Mut_*kdr*_F	Forward	TCCGGAATTGGAGAAGGTGC	20	55	Sequencing	This study
4Mut_*kdr*_R	Reverse	TCAAGCCATCGCCCATGATT	20	55	Sequencing	This study

*kdr* Knockdown resistance, *PASA *PCR amplification of specific alleles

**Fig. 2:**
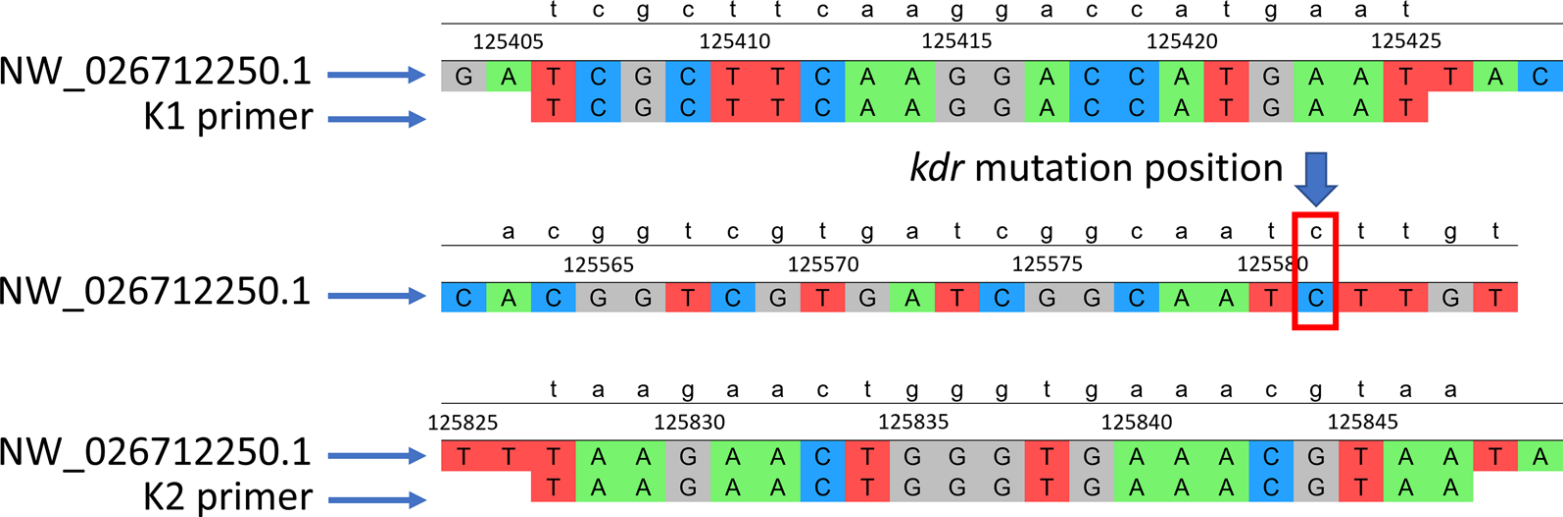
Primers K1 and K2 bind to the voltage-sensitive sodium channel of the house fly *M. domestica* and produce a 448-bp segment that encompasses the *kdr* mutation L1014F at position number 125581 compared to GenBank accession number NW_026712250.1. *kdr*, Knockdown resistance

A secondary method was employed to establish the genotypes of house flies, involving PCR amplification of house fly DNA using the K1 and K2 primers (Table 1); the PCR conditions mirrored those detailed by Mazzoni et al. [47]. The amplified products then underwent Sanger sequencing using the forward and reverse primers. UGENE software was used to analyze the DNA sequences [48], and the chromatograms were reviewed using SnapGene software (www.snapgene.com). Within the UGENE software, the sequences underwent multiple alignment with the Clustal Omega algorithm, facilitating precise comparison to the *Vssc* gene of the house fly available on the National Center for Biotechnology Information (NCBI) database (GenBank accession number NW_026712250.1). This alignment served as a foundational step, enabling the identification of the specific position of the *kdr* mutation (Fig. 2). Furthermore, the DNA sequence ACGGTCGTGATCGGCAATT was used as a guiding reference sequence in the multiple alignments for pinpointing the genetic variation (*kdr* mutation) in each of the house fly sequences from this study. In this context, susceptible homozygous (SS) insects have CTT, heterozygous (RS) insects have YTT, where Y is C/T, and resistant homozygous (RR) insects have TTT. The files generated from the sequencing process were uploaded to SnapGene to determine the shape and quality scores of the chromatograms, revealing two peaks of CC in SS insects, two peaks of TT in RR insects and two peaks of CT in RS insects at the correct *kdr* mutation position.

**Fig. 3:**
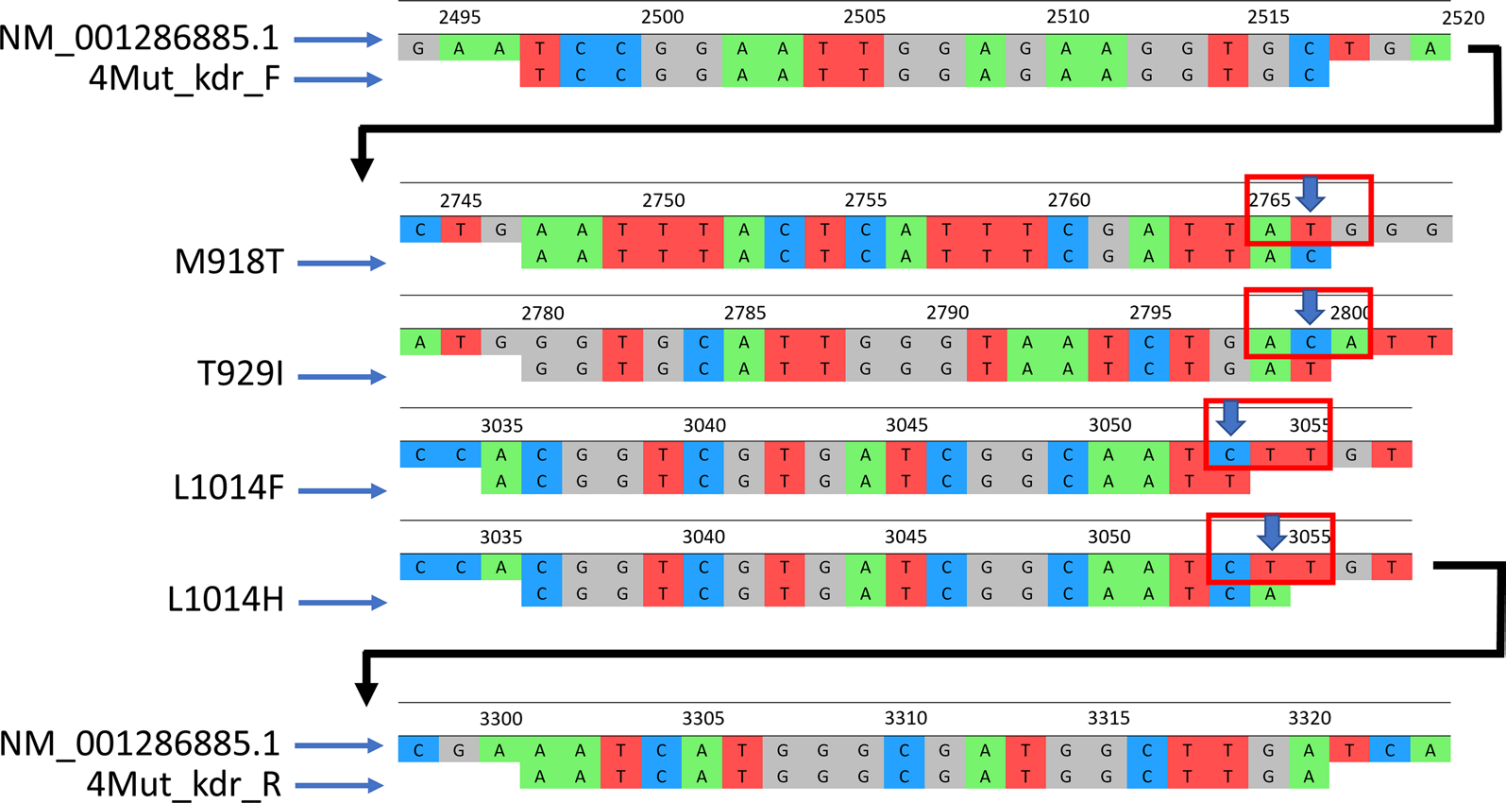
The primer pair 4Mut_*kdr*_F/R detects the presence of four *kdr* mutations (M918T, T929I, L1014F and L1014H) using complementary DNA as a template in reverse-transcriptase-PCR. The position of each mutation on the *Vssc* gene is marked with an arrow, and the DNA sequences used as guiding sequences (M918T, T929I, L1014F, L1014H) for easy mutation detection are shown.* kdr*, Knockdown resistance; *Vssc*, voltage-sensitive sodium channel gene

As mentioned earlier, the *kdr* mutation L1014F can be detected using PASA on a gel or by applying the primers K1 and K2 via DNA sequencing. However, as with sequencing, it is challenging to detect this mutation and the *super-kdr* mutation simultaneously because a large intron (approximately 1700 bp) separates these two mutations on gDNA. Therefore, this difficulty is overcome using complementary DNA (cDNA) which does not contain introns. Thus, we designed the primer pair 4Mut_*kdr*_F/R (Table 1) that anneals to the cDNA and amplifies a region that includes four *kdr* mutations (M918T, T929I, L1014F and L1014H). RT-PCR was performed using the OneStep RT-PCR Kit (Qiagen) following the manufacturer’s instructions. The thermocycling conditions were reverse transcription at 50 °C for 30 min; denaturation at 95 °C for 5 min; followed by 35 cycles of 95 °C for 30 s, 55 °C for 30 s and 72 °C for 60 s; with a final extension at 72 °C for 10 min. The PCR products were checked by electrophoresis in an agarose gel (1.5%). All PCR products were sequenced using the Sanger method. Sequence alignment was performed with the coding sodium channel protein para-like (para-like) mRNA sequence (GenBank accession number NM_001286885.1). Four DNA fragments were used as guiding references in the multiple alignment for pinpointing the genetic variation of each *kdr* mutation (M918T: AATTTACTCATTTCGATTAC; T929I: GGTGCATTGGGTAATCTGAT; L1014F: ACGGTCGTGATCGGCAATT; L1014H: CGGTCGTGATCGGCAATCA) (Fig. 3). All PCR amplifications were performed in a Swift Max Pro thermocycler (ESCO, Singapore). A negative control containing no template was included in every PCR assay to ensure no contamination. All Sanger sequencing procedures for this study were conducted at the Genomic Unit of the Biology Department at UAE University.

### CDC bottle bioassay

We used the active ingredient of the insecticide deltamethrin PESTANAL® (Sigma-Aldrich, St. Louis, MO, USA) and followed the CDC bottle bioassay protocol [44]. In brief, a specific weight (mg) of deltamethrin was dissolved in a known volume (ml) of acetone to produce a stock solution. Then, several serial dilutions (working solutions) were prepared to be used in determining the diagnostic dose and time using susceptible house flies. We acquired susceptible house flies from Dubai Municipality, UAE; these had been bred in the laboratory and not exposed to insecticides for 15 years, and we subsequently reared them in the laboratory under standard house fly rearing conditions. For each CDC bioassay, we used five standard 250-ml DURAN® (DWK Life Sciences GmbH, Mainz, Germany) glass bottles with a screw cap. The inside surfaces of four bottles were coated with 1 ml of deltamethrin working solution at the appropriate concentration; the fifth bottle was treated with acetone only and served as a control. In each bottle, we placed 25 flies and monitored mortality at 15-min intervals up to 2 h (time points: 0, 15, 30, 45, 60, 75, 90, 105 and 120 min). After determining the diagnostic dose and time using these susceptible flies, we tested field-collected flies and determined the level of resistance.

### Statistical analysis

The PASA results were processed in a Microsoft Excel spreadsheet (Microsoft Corp., Redmond, WA, USA) to compute averages and percentages for each house fly genotype. Hardy–Weinberg Equilibrium (HWE) was determined using an online tool ([49]; http://apps.biocompute.org.uk/hwe-mr-calc.html), with the Chi-square (*χ*^2^) and *P*-value calculated.

## Results

**Fig. 4:**
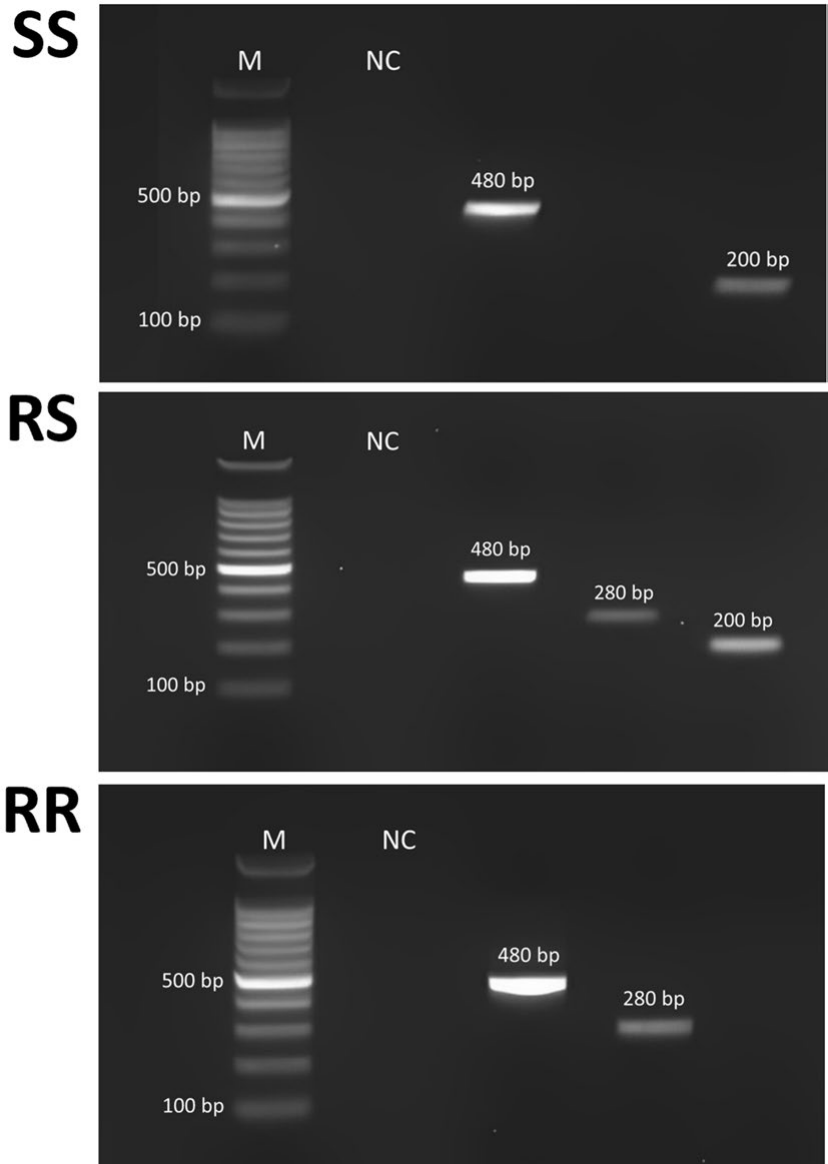
Genotyping of house fly *Musca domestica* based on the *kdr* mutation L1014F using PASA and agarose gel (1.5%) stained with ethidium bromide. Susceptible insects (SS; *sus*/*sus*) had 480-bp (control band) and 200-bp bands. Heterozygous insects (RS; *kdr*/*sus*) had 480-, 280- and 200-bp bands. Resistant homozygous insect (RR;* kdr*/*kdr*) has 480- and 280-bp bands.* kdr*, Knockdown resistance; M, 100-bp marker (Promega, Madison, WI, USA); NC, negative control; PASA, PCR amplification of specific alleles

**Fig. 5:**
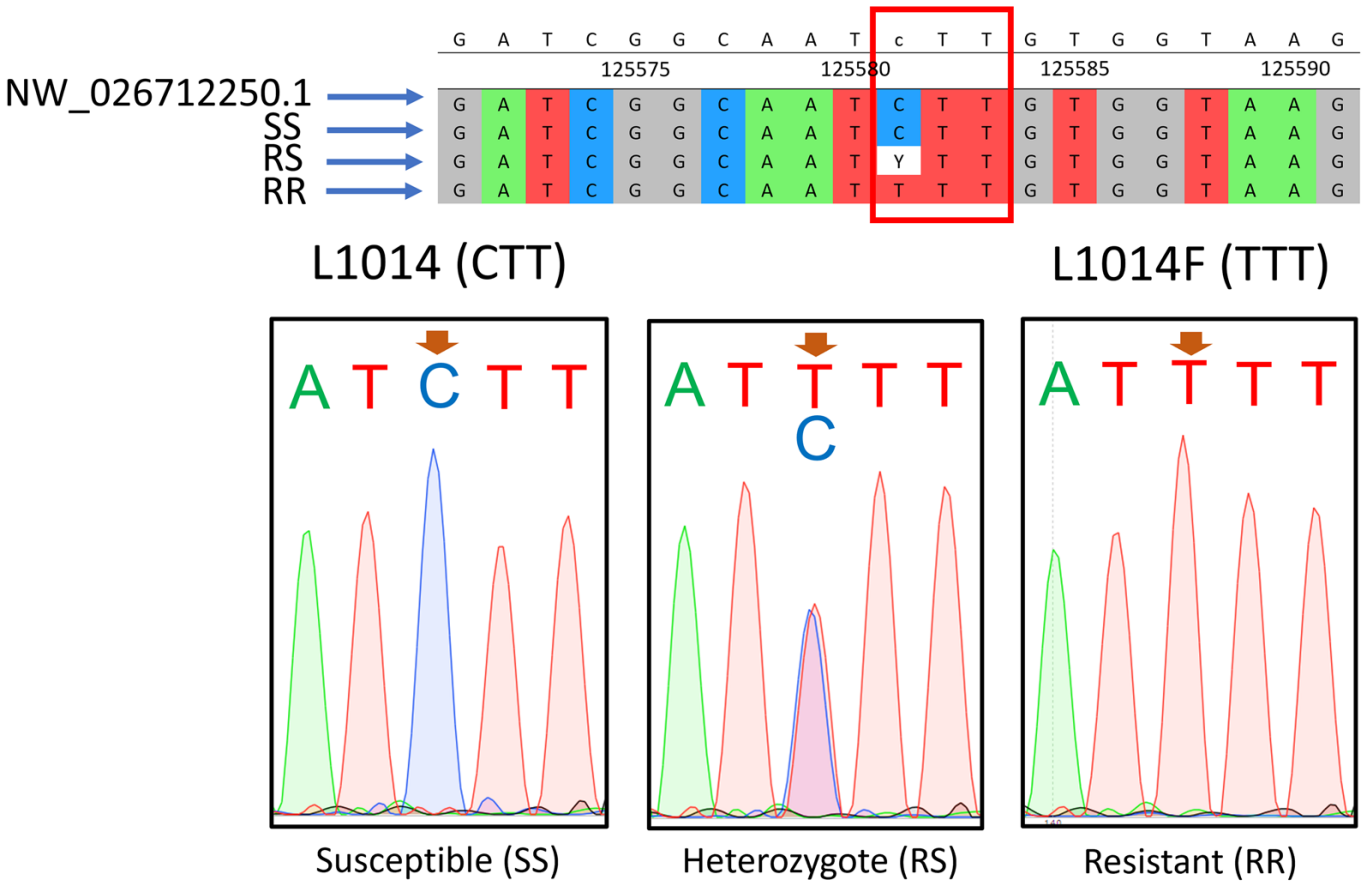
Genotyping of the house fly *Musca domestica* based on the *kdr* mutation L1014F using DNA sequencing. Sequences are aligned with the genomic reference sequence (GenBank accession number NW_026712250.1) using Unipro UGENE software. The chromatograms were viewed using SnapGene software (www.snapgene.com). The mutation occurs at position number 125581. Susceptible (SS) insects have the sequence CTT; heterozygous (RS) insects have the sequence YTT, where Y is C/T; and resistant homozygous (RR) insects have the sequence TTT. Chromatograms show a single peak in SS and RR genotypes and two peaks in RS genotypes at the *kdr* mutation position.* kdr*, Knockdown resistance; RR, homozygous resistant (*kdr*/*kdr*);* RS*, heterozygous (*kdr*/*sus*); SS, homozygous susceptible (*sus*/*sus*)

The primers used in PASA analysis successfully amplified the three target bands (480, 280 and 200 bp) in the agarose gel (Fig. 4). The *kdr* allele was detected in house flies collected from 18 of the 19 sampled locations (Table 1). The only exception was Remah, where the *kdr* allele was not found. The highest percentage of house flies with the *kdr* allele (46.9%) was observed in Al Rawdah and Al Shuwaymah, and the lowest level (9.4%) was detected in house flies from Shahama City and Baniyas. Overall, the homozygous resistant genotype (RR) was identified in 14 insects, constituting 5.0% of the house fly population, while the heterozygous genotype (RS) was present in 102 insects, accounting for 36.5% of the house fly population. In terms of geographic distribution, the RR genotype was prevalent in 52.6% of the study locations (10/19) (Table 2). In addition, all the tested house fly populations were in HWE (*P*-value of *χ*^2^ > 0.05), with the exception of those from Hadbat Al Zafranah, Al Danah and Al Shuwaymah) (Table 2). The K1 and K2 primers were successfully used in the PCR assays, producing the target band of 448 bp. These same two primers were used in Sanger sequencing, with the results confirming the PASA data regarding the *kdr* genotypes with L1014F observed in the studied house fly populations and revealing the presence of an additional mutation, L1014H. The *kdr* mutation L1014F was identified at position number 125581 (Fig. 2) according to the reference sequence (GenBank accession number NW_026712250.1). In susceptible (SS) insects, the sequence was CTT, while heterozygous (RS) insects had the sequence YTT, where Y represents C/T; resistant homozygous (RR) insects exhibited TTT (Fig. 5). The chromatograms displayed a single peak in SS and RR insects and two peaks in RS insects at the *kdr* mutation position. The primer pair 4_Mut_*kdr*_F/R, designed specifically for this study, amplified the target region containing four *kdr* mutations (M918T, T929I, L1014F and L1014H) and revealed the presence of a second new mutation, T929I, in the tested house flies. The CDC bottle bioassays on susceptible house flies revealed that the diagnostic time and diagnostic dose for deltamethrin was 60 min and 4.5 ug/ml, respectively. Accordingly, deltamethrin resistance was detected in house flies from two locations, Al Shwaib and Al Aflaj, out of 16 locations tested (12.5%) (Table 3).

**Table 2:** Distribution of the knockdown resistance mutation L1014F, percentage, and Chi-square of Hardy–Weinberg equilibrium in the sampled *Musca domestica* populations based on results of the PCR amplification of specific alleles analysis, in Abu Dhabi, UAE, 2023

Location map code^a^	Location name^a^	*N* house flies tested	N flies with genotypes^b^:			% RR alleles	*χ*^2^	*P*
			RR	SS	RS			
1	Mina Zayed	16	1	6	9	34.4	0.9741	0.3236
2	Hadbat Al Zafranah	6	0	0	6	18.8	6	0.0143*
3	Al Danah	19	0	6	13	40.6	5.1376	0.0234*
4	Al Muzoun	15	1	10	4	18.8	0.4166	0.5186
5	Al Mushrif	10	1	6	3	15.6	0.4	0.527
6	Al Bateen	15	0	9	6	18.8	0.9375	0.3329
7	Al Rawdah	17	4	6	7	46.9	0.4623	0.4965
8	Shahama City	18	0	15	3	9.4	0.1487	0.6997
9	Al Samha	18	0	13	5	15.6	0.4682	0.4937
10	Al Bahiya	8	1	3	4	18.8	0.0355	0.8504
11	Baniyas	8	1	6	1	9.4	2.7823	0.0953
12	Mirfa	14	1	9	4	18.8	0.3213	0.5707
13	Madinat Zayed	18	2	9	7	34.4	0.1259	0.7227
14	Al Hayer	9	1	6	2	12.5	1.1479	0.2839
15	Al Khazna	17	0	13	4	12.5	0.3022	0.5824
16	Hili	20	1	15	4	15.6	0.9304	0.3347
17	Remah	8	0	8	0	0.0	-	-
18	Central district	16	0	11	5	15.6	0.5486	0.4588
19	Al Shuwaymah	27	0	12	15	46.9	3.994	0.0456*
	Total	279	14	163	102			

*kdr* Knockdown resistance

*Significantly different at* P* ≤ 0.05, indicating that the population is not in Hardy–Weinberg equilibrium

^a^See Fig. 1 for corresponding codes and sampling locations

^b^*RR* Homozygous resistant (*kdr*/*kdr*),* RS* heterozygous (*kdr*/*sus*), SS homozygous susceptible (*sus*/*sus*)

**Table 3:** Centers of Disease Control and Prevention bottle bioassay using deltamethrin on field-collected house flies

Location	Test date^a^	Insecticide dose (ug/ml)	Resistance status	Time to 100% mortality (min)
Al Rawadah	10 March	4.5	Susceptible	60
Al Mushrif	15 March	4.5	Susceptible	60
Al Bateen	1 April	4.5	Susceptible	60
Al Wathba	1 March	4.5	Susceptible	60
Al Falah	3 March	4.5	Susceptible	60
Yas Island	6 March	4.5	Susceptible	60
Mussafah Shabiya	8 March	4.5	Susceptible	60
Al Adlah	10 March	4.5	Susceptible	60
Madinat Zayed	16 March	4.5	Susceptible	60
Bida Bint Saud	17 April	4.5	Susceptible	60
Al Shwaib	28 April	4.5	Resistant	90
Al Saa	27 March	4.5	Susceptible	60
Bu Kirayyah	1 March	4.5	Susceptible	60
Malaqit	14 March	4.5	Susceptible	60
Al Rawdah Al Sharqiyah	11 April	4.5	Susceptible	60
Al Aflaj	14 April	4.5	Resistant	90

Mortality was checked at 15-min intervals post assay initiation, up to 2 h (0, 15, 30, 45, 60, 75, 90, 105 and 120 min)

^a^All test dates were in 2023

## Discussion

Repeated use of pyrethroid insecticides can drive the selection for mutations or amino acid substitutions in the house fly VSSC, causing resistance in target insects [50]. The putative pyrethroid binding pocket on the VSSC may experience a decrease in insecticide affinity due to the selected mutations, resulting in target-site insensitivity [51]. Accordingly, the insecticide-induced selection pressure would allow those insects carrying resistance genes to survive insecticide treatment and pass on the resistance trait to their offspring, thereby driving an increase in the frequency of the resistance allele within the population over time [52, 53]. The development of resistance against pyrethroids has been documented in various insects worldwide, including mosquito species such as *Anopheles gambiae* sensu stricto [54] and *Anopheles arabiensis* [55], as well as the house fly *M. domestica* [39]. In the present study, we confirmed the presence of the previously known *kdr* mutation L1014F, which was reported in the UAE in 2014, with our assays revealing its presence in most of the house fly populations tested in Abu Dhabi in 2023. In addition, we also recorded the presence of two new *kdr* mutations, L1014H and T929I. We also described *kdr* mutation detection methods based on DNA sequencing and set up a CDC bottle bioassay to assess deltamethrin resistance in house flies in the UAE.

The successful use of PASA in this study shows that it is a simple method for screening house fly populations for the presence of *kdr* mutation L1014F. Our findings revealed that all sampled populations had the *kdr* allele, with the exception of a single population. The widespread occurrence of the resistance allele strongly suggests that resistance to pyrethroid insecticides has steadily increased over time in the house fly populations examined. Additionally, the spread of the resistance allele may suggest that house fly populations face consistent insecticide selection pressures [56] from pyrethroid pesticides or significant fly movement [57] between the geographical regions in the study area. In certain areas, such as Al Rawdah and Al Shuwaymah, the percentage of the *kdr* allele in the house fly populations reached nearly 50%, whereas in the other regions, it was remarkably lower. It should be noted that the RR genotype was found to be predominant in house flies from 52.6% of the surveyed locations, covering 10 out of 19 sites, and this distribution can increase resistance frequency. The RR genotype possesses two copies of the resistance allele, resulting in high insecticide resistance. Upon surviving exposure to insecticides, these RR insects can transmit both copies of the resistance allele to their offspring, thereby increasing the prevalence of resistance alleles within the population. As resistance alleles spread, insecticides targeting susceptible alleles lose effectiveness, necessitating alternative control methods. However, if susceptible individuals migrate into a new area, they can dilute the resistance in that area [58]. Further, when RR insects are present, the spread of resistance among the population can increase because RR insects can mate with susceptible insects (SS) and produce RS offspring [59]. As RS offspring still carry one copy of the resistance allele, they can contribute to resistance development [60] and, as such, help sustain and spread resistance. In some cases, additional resistance mechanisms may be selected by the presence of RR insects, resulting in super-resistant insects [61] that are even more difficult to manage. Moreover, controlling RR insects often requires additional alternative pest control strategies. Thus, the application of pyrethroid insecticides can affect the genetic variation in a population, which will remain constant from one generation to the next without disruptive factors. In this study, only three populations showed significant deviation from HWE, which indicates that some evolutionary force, such as mutation, selection, genetic drift or non-random mating [62, 63], may be acting on the population, causing the observed genotype frequencies to deviate from the expected frequencies.

One of the advantages of PASA is that it can be performed quickly in any laboratory equipped with a PCR apparatus, an agarose gel electrophoresis unit and a gel documentation system. However, the wide use of Sanger sequencing and its lower costs make detecting *kdr* mutations a more appealing and accurate choice when studying resistance in insect populations. In the present study, we mainly used PASA to detect the L1014F mutation and utilized Sanger sequencing only to confirm the presence of this mutation in house flies. Based on sequencing, the mutation was easily detected using a multiple sequence alignment tool, such as MultAlin (http://multalin.toulouse.inra.fr/multalin/) [64]. Susceptible insects (SS) had the CTT sequence; heterozygous insects (RS) had the YTT sequence, with Y representing C/T; and the resistant homozygous insects (RR) had the TTT sequence. Therefore, for future studies, we recommend using sequencing over PASA in laboratories that have the knowledge, equipment and finances for sequencing. Several studies have reported using sequencing for *kdr* mutation detection [41, 47, 65–67]. In short, PASA rapidly detects *kdr* resistance (L1014F) in house flies and is the ideal alternative for laboratories lacking DNA sequencing or needing quick and easy methods.

To date, researchers have identified five mutations in the VSSC of house flies, namely *kdr* (L1014F), *kdr-his* (L1014H), *super-kdr* (M918T + L1014F), *type N* (D600N + M918T + L1014F) and *1B* (T929I + L1014F) [50]. However, up to the present study, only one mutation (L1014F) had been recorded in the UAE, reported for the first time in 2014 [38]. The current study confirms its presence in house fly populations using PCR and DNA sequencing and additionally reports the presence of two more new mutations, namely L1014H and T929I. The persistent presence of the *kdr* allele suggests the need for a UAE-wide insecticide resistance monitoring program, with standardized protocols for collecting and analyzing data on house fly DNA. The present study sheds light on three mutations and can serve as a starting point and a basis for further research on pyrethroid insecticide resistance in house flies in the country. In addition, our study provides a primer pair that can be used to detect four mutations in one RT-PCR assay followed by DNA sequencing. We also suggest that laboratories with the manpower and equipment to work with RNA use the primer pair we designed to detect these four *kdr* mutations using RT-PCR. Genomic DNA can be extracted from house flies for conventional PCR and PASA from both fresh flies and dead flies collected from traps. However, extracting RNA to generate cDNA requires live flies to be collected and then frozen immediately to prevent RNA degradation. This could add an additional level of difficulty to *kdr* detection based on cDNA compared to gDNA. In the current study, we focused only on the Emirate of Abu Dhabi; therefore, we recommend that future studies include flies from all over the UAE to better understand insecticide resistance. Gathering more samples from more areas could uncover the presence of more mutations. Based on published studies, the UAE is one of the few countries in Asia where *kdr* mutations have been found in house flies. So far, they have been detected also in Turkey (*kdr* L1014F and *kdr-his* L1014H) [68], Iran (*kdr-his* L1014H) [65], China (*kdr, super-kdr* and *kdr-his*) [69–71], Japan (*kdr* and *super-kdr*) [72]^72^) and Pakistan (*kdr*) [42]. It should be noted that in the present study, DNA sequencing was employed exclusively to validate the genotypes (RR, RS and SS) identified through PASA, but it was not performed on all the house fly DNA samples due to time and budgetary constraints. Also, we used a relatively small sample size (*n* = 18) when using primers K1, K2 and 4Mut_*kdr*_F/R. The reason for the small sample size was because the purpose of presenting the molecular part in this study was to confirm the results of PASA using K1 and K2 and to demonstrate the feasibility of using one primer pair (4Mut_*kdr*_F/R) for detecting four *kdr* mutations as a foundational tool for prospective resistance studies.

The CDC bottle bioassay is a simple and rapid test to detect insecticide resistance in field-collected insects [73]. Our study provided, for the first time in the UAE, a diagnostic dose and time for deltamethrin tested against susceptible house flies, which is the basis for detecting resistance using the CDC bottle bioassay. Thus, the bioassay reported here will assist future researchers in screening for deltamethrin insecticide resistance in house flies in the UAE. Additionally, our protocol will be a good tool when coupled with Sanger sequencing using the primers designed in this study to detect the four *kdr* mutations. Our CDC bottle bioassay results revealed that 12.5% of the tested house fly populations were resistant to deltamethrin, indicating that resistance should be monitored and managed in these populations. The CDC bioassay will help to perform resistance monitoring and gain a better understanding of resistance development in house flies. While the CDC bottle bioassay has been mainly deployed for mosquitoes, several studies have reported its use on adult house flies to test the synergistic effect of insecticides [74] and study the impacts of three ethnobotanical culinary plants [75]. One of the additional advantages of the CDC test is that it uses 100 insects from each sampling site, with four replications of 25 insects each. This means that its results are based on a large number of insects, unlike a DNA-based resistance detection test, which typically uses fewer insects per sampling site. Further, the CDC bottle bioassay does not require sophisticated equipment and is much cheaper than molecular tests involving DNA or RNA extraction, PCR, gel electrophoresis and sequencing. Thus, we suggest using an integrative approach to work on insecticide resistance in house flies in the UAE. This would initially involve using the CDC bottle bioassay for field screening, then PASA and DNA sequencing to identify the types of mutations in the resistant house fly populations.

Controlling house fly populations through the sole use of insecticides can drive not only the development of even higher levels of resistance but also environmental chemical pollution, both of which are detrimental to human health. As an alternative, employing an integrated pest management (IPM) program, which takes a holistic approach to managing house fly populations, including a combination of biological, mechanical and cultural strategies, is an effective and environmentally friendly option [76, 77]. In general, the establishment of an IPM program for house flies is urgently needed in the UAE. To ensure a successful IPM plan, it is imperative to monitor susceptibility to pyrethroids, which can help in the detection of resistance at an early stage and predict the evolution and spread of resistant phenotypes and genotypes [50]. Thus, before resorting to insecticide use, it is important to exhaust all nonchemical methods first. Further, it should be noted that the lifespan of insecticide products can be influenced by the strategies employed in their application [61] because the development of resistance can lead to insecticide failure. Our findings enable UAE researchers to screen for deltamethrin resistance in house flies, thereby enhancing global knowledge. The detection of *kdr* mutations suggests that neighboring countries should also conduct screenings and study fly movement to understand the spread of resistance.

## Conclusions

Using DNA sequencing, we confirmed the presence of a known *kdr* mutation and uncovered two new *kdr* mutations in house flies from Abu Dhabi. Additionally, we detected deltamethrin resistance in these flies using a CDC bottle bioassay. Further research is recommended to comprehensively identify more *kdr* mutations in UAE house fly populations and assess their impacts on control strategies.

## Data Availability

The DNA sequences produced by this study are available for download from the open-access Zenodo repository at https://doi.org/10.5281/zenodo.10013574.

## Abbreviations

CDC: Centers for Disease Control and Prevention
cDNA: Complementary DNA
gDNA: Genomic DNA
HWE: Hardy–Weinberg equilibrium
IPM: Integrated pest management
*kdr*: Knockdown resistance gene
NCBI: National Center for Biotechnology Information
PASA: PCR amplification of specific alleles
RT-PCR: Reverse transcriptase-PCR
UAE: United Arab Emirates
VSSC: Voltage-sensitive sodium channel
